# Comparative analysis of the therapeutic efficacy of remimazolam tosylate and propofol in older adults undergoing painless endoscopic retrograde cholangiopancreatography

**DOI:** 10.3389/fphar.2024.1404536

**Published:** 2024-10-17

**Authors:** Yu-quan Tian, Di-kun Chen, He-ming Zhang, Yong-mei Sun

**Affiliations:** ^1^ Department of Anesthesiology, Shandong Provincial Third Hospital, Jinan, China; ^2^ Department of Anesthesiology, Jinan Seventh People’s Hospital, Jinan, China

**Keywords:** endoscopic retrograde cholangio-pancreatography, hemodynamics, older adults, propofol, recovery time, remimazolam tosylate

## Abstract

**Objective:**

The aim of this study is to conduct a comparative analysis of the therapeutic outcomes associated with the administration of remimazolam and propofol during painless endoscopic retrograde cholangiopancreatography (ERCP) procedures in older adults.

**Methods:**

A total of 140 older adults who underwent elective painless ERCP were randomly assigned to two groups using the random number table method: the remimazolam group and the propofol group, each consisting of 70 patients. In the remimazolam group, anesthesia was administered using a combination of remimazolam and opioids, while in the propofol group, a combination of propofol and opioids was used. Comparative assessments between the two groups included anesthesia induction time, first induction success rate, intraoperative hemodynamics, awakening duration, stress response index, and the incidence of adverse reactions.

**Results:**

The remimazolam group exhibited a prolonged anesthesia induction time compared to the propofol group and a lower success rate of first induction (*P* < 0.05). At the point of endoscope entry (T2) and 10 min post-operation (T3), patients in the remimazolam group demonstrated higher mean arterial pressure (MAP), heart rate (HR), and bispectral index (BIS) values compared to those in the propofol group (*P* < 0.05). Furthermore, the remimazolam group had shorter durations for eye-opening, consciousness recovery, and residence in the recovery room compared to the propofol group *(P* < 0.05). Post-surgery levels of epinephrine (E), norepinephrine (NE), and cortisol (Cor) at 24 h were lower in the remimazolam group than in the propofol group (*P* < 0.05). The incidence of adverse reactions was significantly lower in the remimazolam group (18.57%) compared to the propofol group (31.43%) (*P* < 0.05).

**Conclusion:**

Remimazolam exhibits a longer induction time compared to propofol in the painless diagnosis and treatment of ERCP in older adults. However, it provides a more stable circulatory state post-induction and throughout the operation, reduces stress response, enables rapid recovery, and has a lower incidence of serious adverse reactions. These attributes suggest that remimazolam has potential for widespread clinical application and adoption.

**Clinical Trial Registration:**

clinicaltrials.gov, identifier ChiCTR2400080926.

## 1 Introduction

Endoscopic retrograde cholangiopancreatography (ERCP) is a widely utilized therapeutic method for managing biliary and pancreatic diseases, known for its minimally invasive nature and surgical precision. The procedure involves inserting a duodenoscope into the descending duodenum via the oral cavity. Surgical instruments or an ultra-fine cholangioscope are then introduced through the duodenal papilla, providing access to the bile duct and pancreatic duct. Diagnosis and treatment are achieved through the injection of a contrast medium under X-ray guidance. ERCP is routinely used for the management of conditions such as bile duct stones, bile duct strictures, pancreatitis, pancreatic cysts, and other related diseases. (Tagawa et al., 2021; Ribeiro et al., 2021).

Despite its minimally invasive nature, the traction stimulation during ERCP can cause trauma to patients, leading to intraoperative hemodynamic abnormalities that may impede the smooth progression of the procedure. Additionally, the substantial postoperative stress response in patients undergoing ERCP contributes to significant postoperative pain, hindering normal recovery. (Dişçi et al., 2022; Zhang et al., 2021).

Addressing these challenges requires the development of a tailored anesthesia plan for patients undergoing ERCP. Effective anesthesia methods are crucial to mitigate hemodynamic fluctuations during surgery, reduce intraoperative and postoperative stress responses, and reduce postoperative pain. (Ijaz et al., 2021). In recent years, anesthesia for ERCP has predominantly relied on the administration of propofol in combination with sufentanil. Although this approach achieves satisfactory anesthesia depth, it presents issues such as suboptimal intraoperative hemodynamic stability, prolonged anesthesia recovery time, and pronounced intraoperative and postoperative stress responses. (Tao et al., 2022).

A promising alternative is remimazolam tosylate, a novel sedative anesthetic with potent analgesic and sedative effects, rapid onset, and stable maintenance. Remimazolam tosylate has demonstrated utility in painless diagnostics and examinations, general intravenous anesthesia, as well as local anesthetic analgesia and sedation. (Díez Ruiz et al., 2023).

Physiological changes in the cardiovascular system among older adults reduce cardiac function, resulting in decreased tolerance to intense stimuli. Age-related changes in the respiratory system lead to diminished lung ventilation and gas exchange efficiency, as well as impaired respiratory regulation. These changes increase the susceptibility of older adults to post-anesthesia hypoxemia, potentially resulting in complications such as arrhythmias. Furthermore, declining liver and kidney function impacts the clearance of medications that rely on hepatic metabolism and renal excretion. Consequently, the careful selection of safe and stable medications is particularly important for older adults undergoing ERCP.

Therefore, the focus of this study is on older adults undergoing painless ERCP, with the objective of comparing and analyzing the effects of remimazolam tosylate and propofol during and after the procedure.

## 2 Research methodology

### 2.1 Study participants

The study included a total of 140 older adults who underwent painless ERCP at the Third Hospital of Shandong Province from October 2021 to October 2023. Participants were randomly assigned to either the remimazolam group or the propofol group, with each group consisting of 70 patients. No statistical differences in baseline characteristics were observed between the two groups (*p* > 0.05) (Table 1).

**TABLE 1:** Comparison of the general data between the two groups.

Groups	Cases	Sex		Age (years)	BMI(kg/m^2^)	ASA grade		Operation time (min)
		Male	Female			Grade I	Grade II	
Remimazolam group	70	40	30	78.11 4.00	23.1 ± 2.74	19	51	36.54 ± 6.22
Propofol group	70	39	31	78 ± 3.63	23 ± 2.74	19	51	36.8 ± 5.58
*t/χ2* value		0.029^a^		0.177^b^	0.216^b^	0.000^a^		−0.258^b^
*p*-value		0.865		0.860	0.829	1.000		0.797

^a^ chi-square test.

^b^ independent t-test.

The inclusion criteria were as follows: (1) Patients with a confirmed diagnosis of biliary and pancreatic disease; (2) Patients requiring ERCP surgery with appropriate surgical indications; (3) Patients categorized as ASA grade I or II; and (4) Patients with no contraindications or allergic reactions to remimazolam tosylate, propofol, or other anesthetic drugs.

The exclusion criteria were as follows: (1) Patients with severe cardiovascular or neurological disease; (2) Patients with contraindications to surgery; (3) Patients with known allergies to benzodiazepines and opioids, or with prolonged use of such drugs; (4) Patients with severely abnormal liver and kidney function; and (5) Patients with incomplete data collection of observation indicators.

Informed consent was obtained from all participants and ethical approval was obtained for the implementation of the research protocol.

### 2.2 Sample size calculation

The primary objective of this study is to compare the therapeutic effects of propofol and remimazolam tosylate in older adults undergoing painless ERCP. The anticipated sample size calculation is as follows:

Given a Type I error rate (α) of 0.05 for a two-tailed test, a Type II error rate (β) of 0.2, corresponding to a power of 0.8 (1 - β), and assuming equal group sizes, effect size is calculated using Cohen’s (2016) recommendations. A moderate effect size of Cohen’s d = 0.5 is used as the basis for the calculation. The sample size formula is as follows:

n=2∗Z1−α2+Z1−β2d2=2∗1.96+.84220.52=2∗2.80220.25≈62.776

Therefore, 70 patients were selected from each of the two groups in this study.

### 2.3 Anesthesia methods

Patients in both groups underwent an 8-h fasting period coupled and a 2-h period of water deprivation. Upon entering the operation room, normal saline was uniformly administered, and patients were positioned in the left prone posture. Thin pads were placed under the armpits of the patients and the right knee to ensure airway patency. Oxygen was administered via a nasal cannula (2–3 L/min) until the patients left the recovery room. A blood pressure cuff was applied to the left upper limb, and an anesthesia monitor was connected to continuously monitor the electrocardiogram, non-invasive blood pressure, respiratory rate, pulse oximeter oxygen saturation (SpO2), and bispectral index (BIS). Before anesthesia induction, first-aid drugs and endotracheal intubation equipment were prepared as standard procedure. Following a 5-min resting period, both groups received a 5 μg injection of sufentanil citrate (Yichang Renfu Pharmaceutical, Approval Number H20054171, specification: 1 mL: 50 μg) to initiate induction.

In the remimazolam group, patients were intravenously administered 0.2 mg/kg of remimazolam tosylate (Jiangsu Hengrui Pharmaceutical, Approval Number H20190034, specification: 36 mg) after 1 min. Endoscopic treatment commenced when the modified observer assessment of the alert/sedation (MOAA/S) score was ≤2 points. If patients exhibited body movement and noticeable swallowing motions, an additional 2.5 mg of remimazolam tosylate was administered each time.

Anesthesia depth was maintained with remimazolam tosylate in combination with remifentanil (Yichang Renfu Pharmaceutical, Approval Number H20030197, specification: 1 mg).

In the propofol group, patients received an intravenous injection of 1.5 mg/kg of propofol emulsion (Beijing Fresenius Kabi Medicine, specification: 20 mL: 200 mg, Approval Number HJ20170306). Endoscopic treatment commenced when the MOAA/S score was ≤2 points. If patients displayed body movement and evident swallowing motions, an additional 0.5 mg/kg of propofol injection was administered. Anesthesia depth was maintained with propofol in combination with remifentanil.

Post-operatively, patients in the remimazolam group received 0.3 mg of flumazenil (Hunan Zhengqing Pharmaceutical, Approval Number H20133014, specification: 10 mL: 1 mg) via intravenous injection to counteract the residual sedative effects of remimazolam. If oxygen saturation (SpO2) fell below 92% during the operation, immediate measures included lifting the lower jaw, and if the patient ceased breathing, the operation was halted to provide mask-assisted ventilation. Vasoactive drugs were administered if the mean arterial pressure (MAP) fluctuated by more than 25% compared to the measurement after 5 min of rest. For heart rate (HR) below 50 beats/min, 0.3–0.5 mg of atropine sulfate injection (Tianjin Jinyao Pharmaceutical, specification: 1 mL: 0.5 mg, Approval Number H20200382) was administered. All adverse events were promptly treated and documented. Patients were observed in the recovery room for 30 min post-operation and were transferred back to the ward if no abnormalities were detected.

In this study, surgical procedures were conducted by qualified and experienced endoscopists to minimize variability in procedural stimulation. These endoscopists underwent regular specialized training to maintain consistent standards of operation.

### 2.4 Primary and secondary outcomes

#### 2.4.1 Primary outcomes

1) Anesthesia induction time, defined as the duration from the initial administration of anesthesia to achieving a MOAA/S score of ≤2 and successful scope insertion.
2) Awakening time, defined as the duration from the end of the last administration of anesthesia to full recovery.

#### 2.4.2 Secondary outcomes

1) Comparison of hemodynamic indicators: HR and MAP were monitored at specific time points, including after 5 min of rest upon entering the operating room, during endoscope insertion, 10 min after the procedure, and during recovery.
2) Comparison of the incidence of adverse reactions: Statistically compared the incidence of adverse reactions between the two groups, including blood pressure fluctuations (defined as MAP fluctuations exceeding 25% compared to the measurement after 5 min of rest), intraoperative bradycardia (heart rate <50 beats per minute), hypoxemia (SpO_2_ < 92%), and other adverse events.

### 2.5 Evaluation indicators

(1) Anesthesia Induction Index Comparison: Statistical comparisons were made for the duration of anesthesia induction and the success rate of the first induction between the two groups.
(2) Hemodynamic Indicator Comparison: The BeneVision N15 multifunctional ECG monitor (Shenzhen Mindray Biomedical Electronics Co., Ltd.) was used to monitor HR, MAP, and BIS at specific time points: after 5 min of resting upon entering the operation room (T1), at the time of endoscope entry (T2), 10 min after the operation (T3), and at the time of recovery (T4). Comparative analysis of these indicators was conducted between the two groups.
(3) Anesthesia Recovery Time Comparison: The eye-opening time, consciousness recovery time, and residence time in the recovery room were recorded and compared between the two groups.
(4) Postoperative Stress Response Index Comparison: Fasting venous blood samples (3 mL) were collected 24 h before the operation and 24 h post-surgery. Using the PGspin (PRF + CGF) laboratory centrifuge (Ningbo Topsen Scientific Instrument Co., Ltd.) with parameters set at a centrifugation time of 15 min, rotation speed of 3,500 rpm, and a centrifugation radius of 8 cm, the isolated serological specimens were analyzed using enzyme-linked immunosorbent assay. Adrenaline (E), norepinephrine (NE), and cortisol (Cor) levels were detected using the PT3502C microplate detector (Beijing Putian Xinqiao Technology Co., Ltd.), with the kit was procured from Shanghai Xinweiyu Biotechnology Co., Ltd.
(5) Adverse Reaction Incidence Comparison: Statistical comparisons were made for the incidence of adverse reactions, including blood pressure fluctuation (MAP fluctuation exceeding 25% compared to that measured after 5 min of resting), bradycardia (HR < 50 beats/min), hypoxemia (SpO2 < 92%), and cognitive function changes during surgery between the two groups. Sedation depth was assessed using the BIS with a target range of 60–85 to ensure appropriate sedation depth for both groups of patients. During the procedure, the anesthesiologist assessed the anesthesia depth based on three aspects 1) BIS values, 2) monitoring of cardiovascular indicators, including changes in HR and blood pressure, and 3) observation of patient movement in response to varying levels of operative stimuli.

### 2.6 Data analysis

Data analysis was conducted using SPSS 25.0. To assess the normality of the success rate of the initial induction between the two groups, skewness and kurtosis were calculated for continuous variables. For the comparison of hemodynamic indicators, the BeneVision N15 multifunctional ECG monitor (Shenzhen Mindray Biomedical Electronics Co., Ltd.) was used to monitor HR. The results indicated that all variables in this study adhered to a normal distribution. Descriptive statistics were employed, with measurement data presented as mean ± standard deviation (‾χ±s), and counting data expressed as case numbers or percentages [n (%)]. The statistical analysis involved the application of the chi-squared test and *t*-test, with a significance level (α) set at 0.05. *P* < 0.05 was considered statistically significant.

## 3 Results

### 3.1 Comparison of general data

This study included a total of 140 older adults who met the criteria for painless ERCP. No statistically significant differences were observed in age, sex, body weight, ASA grade, operation time, or MAP, HR, and BIS values at the T1 time point between the two groups (*P* > 0.05). These parameters were comparable, as shown in Table 1.

### 3.2 Comparison of intraoperative hemodynamic indicators

Patients in both groups achieved adequate anesthesia depth after two or fewer additional administrations of their respective trial drugs at time point T1. There were no statistically significant differences between the remimazolam group and the propofol group regarding the successful completion of ERCP, as measured by MAP, HR, and BIS (*P* > 0.05). However, at time points T2 and T3, patients in the remimazolam group exhibited significantly higher MAP, HR, and BIS values compared to those in the propofol group (*P* < 0.05). Regarding anesthesia induction time, 38 patients in the remimazolam group achieved successful induction on the first attempt, which was fewer than the 58 patients in the propofol group. Differences in MAP and HR between time points T2, T3, and T4 compared to T1 were not statistically significant in the remimazolam group (*P* > 0.05). In contrast, significant differences in MAP and HR were observed between time points T1 and T2 compared to T3 in the propofol group (*P* < 0.05), indicating smaller hemodynamic fluctuations in the remimazolam group compared to the propofol group, as shown in Table 2.

**TABLE 2:** Comparative analysis of intraoperative hemodynamic indexes (‾χ±s).

Groups	Indexes	Cases	T₁	T₂	T₃	T₄	F	P
Remimazolam Tosylate	MAP (mmHg)	70	86.9 ± 8.84	87.97 ± 8.83	88.51 ± 7.07	88.09 ± 11.95	0.354	0.749
propofol		70	87.09 ± 9.61	71.87 ± 7.28	72.31 ± 5.08	87.97 ± 10.48	75.054	<0.001
*t* value			0.119	11.77	15.567	0.060		
*p*-value			0.905	<0.001	<.001	0.952		
Remimazolam Tosylate	HR (beats/min)	70	75.77 ± 9.58	75.59 ± 10.1	75.87 ± 9.96	75.1 ± 10.99	0.081	0.967
propofol		70	75.8 ± 8.02	67.76 ± 9	67.57 ± 10.33	75.14 ± 11.7	14.570	<0.001
*t* value			0.019	4.860	4.839	0.022	2215.08	<0.001
*p*-value			0.985	<0.001	<0.001	0.982	3544.56	<0.001
Remimazolam Tosylate	BIS	70	97.14 ± 1.59	64.2 ± 4.01	63.99 ± 3.92	95.80 ± 2.51		
propofol		70	97.03 ± 1.68	48.39 ± 5.17	48.39 ± 5.17	93.74 ± 3.57		
*t* value			0.414	20.22	21.70	3.94		
*p*-value			0.68	<0.001	<0.001	<0.001		

MAP: mean artery pressure, HR: heart rate, BIS: bispectral index.

### 3.3 Repeated measures analysis

At time point T1, no significant differences were observed in MAP, HR, or BIS among the groups. MAP, HR, and BIS were measured four times throughout the study. Generalized Estimating Equations (GEE) was used to determine the effects of different drugs, time, and interactions between these variables on the measurements. The GEE model was used to analyze MAP, HR, and BIS with respect to factors including intercept, group (remimazolam besylate vs propofol), time, and group*time interaction. The results are detailed in Table 3.

**TABLE 3:** Testing the effects of Generalized Estimation Equation Models on mean artery pressure, heart rate and bispectral index.

DV	IV	B	SE	Wald χ^2^	*p*-value
mean artery pressure	intercept	79.036	1.435	3034.859	<0.001
	Group^a^	7.807	2.086	14.007	<0.001
	time	0.310	0.530	0.343	0.558
	Group*time	0.100	0.804	0.015	0.901
heart rate	intercept	72.107	1.119	4151.117	<0.001
	Group^a^	3.907	1.850	4.459	0.035
	time	−0.216	0.425	0.257	0.612
	Group*time	0.043	0.699	0.004	0.951
bispectral index	intercept	74.350	0.450	27,300.446	<0.001
	Group^a^	6.993	0.542	166.171	<0.001
	time	−0.996	0.171	34.061	<0.001
	Group*time	0.571	0.203	7.911	0.005

^a^ Remimazolam Tosylate compare with propofol, propofol as reference group.

For MAP, both the intercept (B = 79.036, *P* < 0.001) and HR values (B = 7.807, *P* < 0.05) in the remimazolam group were significantly higher than those in the propofol group. There were no significant differences in MAP and HR between time points T2, T3, and T4 compared to time point T1 in the remimazolam group (*P* > 0.05), and the group*time interaction (B = 0.100, *P* = 0.901) was not significant. Conversely, in the propofol group, significant differences were observed in MAP and HR between the T1 time point and T2 (B = 3.907, *P* = 0.035) and T3 (B = -0.216, *P* = 0.612) time points. The group*time interaction (B = 0.043, *P* = 0.951) was not significant. Regarding the BIS, the intercept (B = 74.350, *P* < 0.001) and BIS values in the remimazolam group were significantly higher (B = 6.993, *P* < 0.001). Time (B = −0.996, *p* < 0.001) and the group*time interaction (B = 0.571, *P* = 0.005) were also significant, indicating that hemodynamic fluctuations in the remimazolam group were comparatively smaller than those in the propofol group.

In summary, compared to propofol, remimazolam besylate significantly influenced all measured variables. Time significantly affected only the BIS in the propofol group, and the group*time interaction was significant only for BIS, as detailed in Table 3.

### 3.4 Comparison of anesthesia indicators, recovery time, and incidence of adverse reactions

Both groups of patients achieved adequate anesthesia depth after receiving two or fewer doses of their respective trial drugs, successfully completing the diagnostic and therapeutic ERCP procedures. However, patients in the remimazolam group experienced longer induction times compared to those in the propofol group. Additionally, the first-time induction success rate was lower in the remimazolam group, with 38 patients achieving successful induction on the first attempt, compared to 58 patients in the propofol group (*P* < 0.05).

As depicted in Table 4, patients in the remimazolam group had shorter eye-opening times, shorter times to consciousness recovery, and shorter recovery room stays compared to those in the propofol group (*P* < 0.05). The incidence of adverse reactions, including blood pressure fluctuations, bradycardia, hypoxemia, and cognitive function changes in the recovery room, was 18.57% (13/70) in the remimazolam group, significantly lower than the 31.43% observed in the propofol group (22/70) (*P* < 0.05), as seen in Table 4. Notably, no severe adverse events, such as the need for endotracheal intubation or postoperative delirium, were reported in either group.

**TABLE 4:** Comparison of anesthesia recovery duration (‾χ±s).

Groups	Cases	Anesthesia induction time (min)	First induction success rate (n/%)	Eye-opening time (min)	Consciousness recovery time (min)	Residence time in the recovery room (min)	Overall incidence of adverse reactions
Remimazolam group	70	2.78 ± 0.78	38 (54.29)	4.23 ± 1.23	5.19 ± 1.22	24.73 ± 2.75	13 (18.57)
Propofol group	70	2.69 ± 0.78	56 (80.00)	9.17 ± 1.51	10.34 ± 1.36	29.91 ± 3.05	22 (31.43)
*t* value		9.988^b^	10.490^a^	21.211	23.61	10.557	4.971
*p*-value		<0.001	<0.001	<0.001	<0.001	<0.001	0.290

^a^ chi-square test. b: independent t-test.

### 3.5 Comparison of postoperative stress response indexes

The stress response indexes, namely, E, NE, and Cor, were significantly lower in the remimazolam group compared to the propofol group at 24 h post-surgery (*P* < 0.05), as presented in Table 5.

**TABLE 5:** Comparison of postoperative stress response indexes (‾χ±s).

Groups	Cases	E (ng/mL)		NE (ng/mL)		Cor (ng/mL)	
		Before operation	24 h post operation	Before operation	24 h post operation	Before operation	24 h post operation
Remimazolam Tosylate	70	23.43 ± 5.06	41.28 ± 5.85	56.33 ± 6.82	65.48 ± 7.86	83.85 ± 10	101.77 ± 12.1
Propofol	70	24.36 ± 5.35	46.18 ± 5.98	57.32 ± 6.68	70.04 ± 8.1	85.09 ± 10.24	110.82 ± 11.23
*t* value		1.052	4.900	0.866	3.376	0.730	4.591
*p*-value		0.295	<0.001	0.388	<0.001	0.467	<0.001

E: adrenaline, NE: norepinephrine, Cor: Cortisol.

## 4 Discussion

Painless ERCP plays a crucial role in the diagnosis and treatment of biliary and pancreatic diseases, characterized by its minimally invasive qualities and surgical precision. However, as an invasive procedure, it inherently generates traumatic stimuli for patients during surgery, resulting in stress reactions such as choking and body movements. These reactions can impede the smooth progression of the diagnostic and treatment processes, potentially causing pronounced hemodynamic fluctuations, complications, and even necessitating the termination of ERCP. Such challenges are detrimental to the postoperative rehabilitation of patients. (Troncone et al., 2022). Therefore, the judicious selection of appropriate anesthesia for painless ERCP in older adults is paramount for the success of the operation.

Over the past few years, general intravenous anesthesia, predominantly based on propofol combined with sufentanil, has been utilized. While this approach exhibits a short half-life and rapid elimination, providing effective anesthesia, it concurrently posed challenges such as significant hemodynamic fluctuations and a heightened stress response. (Weissman et al., 2023). Close monitoring and recording of various vital indicators by the anesthetist in the operating room are essential and yield positive feedback effects.

Remimazolam tosylate, a novel central nervous system depressant belonging to the benzodiazepine class, exhibits potent analgesic and sedative properties, demonstrating favorable effects in both general and local anesthesia contexts. (Guo et al., 2021). In this study, patients in the remimazolam group underwent anesthetic intervention with remimazolam tosylate in combination with sufentanil. In comparison to the propofol group, the remimazolam group revealed a longer anesthesia induction time and a lower success rate of the first induction. The delayed anesthesia onset time in the remimazolam tosylate group is primarily attributed to the longer onset time of remimazolam as compared to propofol. To ensure safety, a small initial dose of remimazolam was administered in this study, while propofol, being rapidly absorbed, contributed to a shorter anesthesia induction time.

In the analysis of hemodynamic indicators, the MAP, HR, and BIS of patients in the remimazolam group at T2 and T3 were higher than those in the propofol group. Moreover, no statistically significant differences were observed in the MAP and HR of patients in the remimazolam group at T2, T3, and T4 compared to those before induction. This indicates that patients treated with remimazolam tosylate for ERCP exhibited greater hemodynamic stability. Remimazolam tosylate, as a potent sedative drug belonging to the novel ester benzodiazepine derivatives, acts on the γ-aminobutyric acid A receptor *in vivo*. This action induces the opening of the central γ-aminobutyric acid A receptor channel, increasing chloride ion influx, hyperpolarizing nerve cell membranes, and inhibiting central neuron activity. As a result, it produces effective analgesic and sedative effects, making it a valuable general intravenous anesthetic drug. (Jang et al., 2022; Park et al., 2020).

In terms of pharmacokinetics, remimazolam tosylate is characterized by rapid onset and lacks inhibitory effects on the cardiovascular system. In comparison to propofol, it exerts less irritation to the blood vessel wall, contributing to stabilized blood pressure, HR, and oxygen saturation, and reduced fluctuations in these hemodynamic indicators. (Hanscom et al., 2022).

In comparing recovery times, the eye-opening time, consciousness recovery time, and recovery room stay for patients in the remimazolam group were shorter than those in the propofol group. This indicates that the application of remimazolam tosylate facilitates the postoperative awakening of patients. The rapid onset of action, swift postoperative recovery, lack of noticeable accumulation in the body, and the absence of toxic side effects such as central inhibition of metabolites contribute to the effectiveness of remimazolam tosylate. Additionally, the inclusion of flumazenil, a remimazolam-specific antagonist, in the anesthetic protocol of this study played an antagonistic role, aiding in the reduction of postoperative recovery time for patients. In contrast, propofol lacks a specific antagonist, underscoring one of the advantages of remimazolam over propofol. Moreover, the potent analgesic and sedative effects of remimazolam tosylate, combined with sufentanil for anesthesia induction and maintenance, lead to a decreased need for additional doses of these drugs during surgery. Consequently, anesthetic drugs can be swiftly eliminated after surgery, resulting in a shortened recovery time for patients. (Chen et al., 2023; Perchoc et al., 2021).

Various factors such as surgical trauma, intraoperative procedures, pain stimulation, and the release of local inflammatory mediators contribute to the upregulation of stress responses in patients, with E, NE, and Cor being common indicators of stress response during surgery. In this study, the levels of E, NE, and Cor in the remimazolam group were lower than those in the propofol group at 24 h after surgery, indicating that the use of remimazolam tosylate could effectively reduce the postoperative stress response in patients. This reduction is attributed to the robust inhibitory effect of remimazolam tosylate on the central nervous system, diminishing the conduction of various nociceptive neuronal impulses and mitigating the stress response induced by surgical site stimulation. (Shi et al., 2020). In the safety assessment, the incidence of adverse reactions in the remimazolam group was lower than that in the propofol group, underscoring the favorable safety profile of remimazolam tosylate.

Previous studies conducted by Li et al. and other ongoing clinical trials have also examined the use of remimazolam. (Li et al., 2024). Key differences between our study and these prior investigations include the type of endoscopic procedure, patient age, and research setting. While most studies focus on gastrointestinal endoscopy, our research examines ERCP, a more complex and invasive procedure that involves the use of a duodenoscope with a larger diameter and greater stiffness, resulting in higher stimulation and longer operation times. Our study specifically targets older adults, approximately 80 years old, as preliminary experiments revealed unsatisfactory results in younger adults who required higher doses of remimazolam and additional opioids. This approach increased the risk of respiratory depression and complicated the surgery. Additionally, the ERCP procedures in our study were conducted in environments with radiation exposure, necessitating more stable anesthesia management.

This research offers valuable insights for anesthesiologists regarding medication choices in diverse clinical scenarios outside the operating room. It highlights the importance of close collaboration between anesthetic nurses and physicians, as timely actions by nurses to manage secretions and prevent complications such as aspiration are key for patient safety. The objective of this study is to establish safer and more effective anesthesia protocols for older adults undergoing ERCP, providing clinical evidence for managing complex cases in high-risk populations.

Several factors influence the outcomes of this study. First, the proficiency of the operating physicians plays a key role, as the level of procedural stimulation can impact patient circulation. To address this, we ensured that qualified endoscopists performed routine procedures, while specialized tasks such as ultrasound examinations were managed by designated specialists. Second, the timing of surgeries throughout the day could affect patient responses and recovery. Third, medication contraindications and precautions, including those related to remimazolam (e.g., allergies to benzodiazepines, myasthenia gravis, schizophrenia), were carefully considered to avoid adverse effects. Fourth, although remimazolam generally has a good safety profile, potential side effects such as dizziness and headaches require vigilant monitoring during clinical use. Finally, due to its similarity to midazolam, remimazolam carries a risk of misuse, necessitating careful oversight in clinical settings. These factors highlight the importance of careful management and tailored approaches within the protocol of the study.

## 5 Limitations

This study has several limitations. First, it was conducted as a single-center case study. Future research should include multi-center trials with larger sample sizes and randomized controlled designs to enhance the generalizability of the findings. Second, the study focused on older adults, who often require safer dosing or fractional administration during anesthesia induction, leading to extended induction times (from the start of medication to achieving the appropriate sedation depth). Further research is needed to examine individualized precision treatments for this age group. Third, while propofol may induce respiratory depression and loss of consciousness by binding to the B3 subunit of GABA receptors, remimazolam interacts with four receptor subtypes involving the B2 subunit. This difference may account for the lower incidence of respiratory depression associated with remimazolam use. However, more detailed investigations are necessary to elucidate the differences between various GABA receptor subtypes and their impact on clinical outcomes.

## 6 Clinical implications

This study underscores the advantages of using remimazolam in older adults. Remimazolam is characterized by its rapid onset and patient recovery, which is especially beneficial for conducting ERCP procedures and reducing patient discomfort and recovery time. It exerts a minimal impact on hemodynamics, which is crucial for older adults who are more vulnerable to hemodynamic fluctuations. Compared to some other sedatives, remimazolam induces lower respiratory depression, thereby lowering the risk of respiratory complications in older adults. Additionally, the sedative effects of remimazolam can be reversed with flumazenil, providing an extra layer of safety, particularly in emergency situations. Remimazolam does not rely on liver and kidney functions for metabolism, making it a potentially safer choice for older adults with hepatic or renal impairments unlike other anesthetics.

Remimazolam also has fewer drug interactions, which reduces the risk of adverse interactions in older adults who may be on multiple medications. The drug allows for dose adjustments based on individual patient conditions, facilitating precision medicine. Furthermore, remimazolam can be used in combination with other drugs to achieve multimodal anesthesia, enhancing anesthetic efficacy while reducing the dosage and side effects associated with single agents. The flexibility and adaptability of remimazolam make it suitable for various clinical settings, including endoscopy rooms, hybrid operating rooms, and outpatient surgical facilities. These attributes position remimazolam as a promising option for providing a safer and more comfortable anesthetic experience for older adults.

## 7 Conclusion

In conclusion, when used for painless ERCP procedures in older adults, remimazolam tosylate demonstrates a longer induction time compared to propofol. However, it offers several advantages over propofol, including more stable hemodynamics post-induction and during the procedure, reduced stress response, rapid recovery, and fewer serious adverse reactions. These benefits highlight the potential for the widespread clinical adoption of remimazolam. Remimazolam is favored due to its rapid awakening, quick patient discharge, alignment with ERAS principles, and its potential to reduce postoperative respiratory and cardiovascular complications such as hypotension compared to propofol. As an ultra-short-acting benzodiazepine, remimazolam provides rapid onset and recovery, mild respiratory and circulatory depression, and minimal impact on organ function and metabolism. These attributes make it a promising option for various clinical scenarios. This study provides theoretical support for the safe use of remimazolam in clinical settings. This approach not only enhances the safety and effectiveness of ERCP in older adults but also offers new insights and methods for enhancing overall medical care for this population, thereby optimizing anesthesia management for older adults undergoing ERCP.

## Data Availability

The original contributions presented in the study are included in the article/supplementary material, further inquiries can be directed to the corresponding author.

## Abbreviations

SBP, Systolic blood pressure; DBP, Diastolic blood pressure; HR, Heart rate; SPO2, Pulse Oxygen saturation; BIS, Bispectral index; BMI, Body mass index; E, Adrenaline; NE, Norepinephrine; Cor, Cortisol; ERCP, Endoscopic Retrograde cholangio-pancreatography; MOAA/S, Modified observer’s assessment of alert/sedation.
